# Surface-Enhanced Raman Spectroscopy of Ammonium Nitrate Using Al Structures, Fabricated by Laser Processing of AlN Ceramic

**DOI:** 10.3390/ma17102254

**Published:** 2024-05-10

**Authors:** Petar Atanasov, Anna Dikovska, Rosen Nikov, Genoveva Atanasova, Katarzyna Grochowska, Jakub Karczewski, Naoki Fukata, Wipakorn Jevasuwan, Nikolay Nedyalkov

**Affiliations:** 1 Institute of Electronics, Bulgarian Academy of Sciences, 72 Tsarigradsko Shosse Blvd., 1784 Sofia, Bulgaria; paatanas@hotmail.com (P.A.); annadikovska8@gmail.com (A.D.); rosen_nikov@abv.bg (R.N.); 2 Institute of General and Inorganic Chemistry, Bulgarian Academy of Sciences, Acad. G. Bonchev Str., Bl. 11, 1113 Sofia, Bulgaria; genoveva@svr.igic.bas.bg; 3 The Szewalski Institute of Fluid-Flow Machinery, Polish Academy of Sciences, 14 Fiszera Street, 80-231 Gdansk, Poland; kgrochowska@imp.gda.pl; 4 Faculty of Applied Physics and Mathematics, Institute of Nanotechnology and Materials Engineering, Gdańsk University of Technology, Narutowicza 11/12 Street, 80-233 Gdańsk, Poland; jakkarcz@pg.edu.pl; 5 Research Center for Materials Nanoarchitectonics (MANA), National Institute for Materials Science (NIMS), 1-1 Namiki, Tsukuba 305-0044, Japan; fukata.naoki@nims.go.jp (N.F.); jevasuwan.wipakorn@nims.go.jp (W.J.)

**Keywords:** laser surface structuring, aluminium nanostructures, SERS, detection of nitrates

## Abstract

This work presents results on laser-induced surface structuring of AlN ceramic and its application in Surface-Enhanced Raman Spectroscopy (SERS). The laser processing is performed by nanosecond pulses in air and vacuum. Depending on the processing conditions, different surface morphology can be obtained. The ablation process is realized by ceramic decomposition as the formation of an aluminium layer is detected. The efficiency of the fabricated structures as active substrates in SERS is estimated by the ability of the detection of ammonium nitrate (NH_4_NO_3_). It is conducted for Raman spectrometer systems that operate at wavelengths of 514 and 785 nm where the most common commercial systems work. The obtained structures contribute to enhancement of the Raman signal at both wavelengths, as the efficiency is higher for excitation at 514 nm. The limit of detection (LOD) of ammonium nitrate is estimated to be below the maximum allowed value in drinking water. The analysis of the obtained results was based on the calculations of the near field enhancement at different conditions based on Finite Difference Time Domain simulation and the extinction spectra calculations based on Generalized Mie scattering theory. The structures considered in these simulations were taken from the SEM images of the real samples. The oxidation issue of the ablated surface was studied by X-ray photoelectron spectroscopy. The presented results indicated that laser structuring of AlN ceramics is a way for fabrication of Al structures with specific near-field properties that can be used for the detection of substances with high social impact.

## 1. Introduction

Surface-Enhanced Raman Spectroscopy (SERS) is a powerful method for the detection and analysis of different materials [1,2,3]. It is based on specific properties of nanostructures that lead to the strong enhancement of the Raman signal of material that is in close vicinity to the nanostructure. Two main mechanisms are responsible for this enhancement: (i) a local increase in the electric field intensity; and (ii) charge transfer between the analysed material and the nanostructure that contributed to stronger excitation. The studies performed indicate that the contribution of the first mechanism is larger [4]. The efficiency of the enhancement of the electric field in the near-field zone of a metal nanostructure strongly depends on the efficiency of plasmon excitation—collective oscillations of the electron system. The most explored plasmonic properties are based on the nanostructures, which involves gold and silver. These noble metals have resonances in the near UV and visible spectral regions, where commercial laser sources, that are usually used for excitation, are well developed. The number of materials used in plasmonics is increasing rapidly in the search of cheaper alternatives and expanding the wavelength range of applications [5]. Recently, attention has been focused on aluminium as a material with promising plasmonic properties [6,7]. One reason for the interest in Al plasmonics is that aluminium’s plasma frequency is higher than that of Au and Ag and this allows for significant surface plasmon responses to occur in the UV spectral range. Moreover, Al is cheaper than noble metals and technologies for nanostructure fabrication can be efficiently applied for this metal. It is a stable metal, due to the formation of a self-limiting native oxide layer protecting the metal surface from further oxidation and from contaminants. Furthermore, in case of Al, the surface plasmon resonances can sustain at wavelengths higher or shorter than the interband transition, which is situated at 800 nm [8]. This means that aluminium maintains its optical properties, related to plasmon excitation, over a broader range than Ag and Au. Consequently, Al nanostructure plasmonic properties can be used successfully in visible spectra except the ultraviolet one. This is demonstrated in application of Al structures in SERS [9,10,11], where efficient detection of different substances is realized for excitation in UV and in the visible spectrum.

The fabrication of nanostructures is still a challenge regarding cost, fabrication speed and reproducibility. Although different methods based on a variety of top-down and bottom-up approaches have been developed, the demonstration of a highly efficient method is still attracting a lot of research efforts. This is because this is crucial for the future of nanotechnology and for the commercialization of potential applications. Laser methods are proven to be able to process any known material with a high spatial resolution [12]. The process of laser ablation, which is generally the removal of material from a target due to laser irradiation, could be a source of nanostructures that can be found in the ablated material, and in addition, can lead to the formation of a variety of nanostructures on the remaining surface. The characteristics of the fabricated materials strongly depend on the laser processing conditions, environment and the processed material. Even this induces a high degree of complexity in the processes involved, and it gives a broad range of ways for tuning the properties of the final structure. The laser radiation can ensure surface modification in specific, desired areas with a high degree of control over the spatial dimensions. Thus, a design of complex systems can be developed [13]. In addition, work in air, without the use of chemicals and multistep protocols also increases the efficiency of the laser approach. Electrochemical methods are efficient alternatives for nanofabrication, but in many cases they require the use of potential environmental pollutants and several steps. Furthermore, laser-based methods may offer better spatial resolution—surface modifications can be easily induced in a micrometre scale. The local laser-matter interaction can also be used as a selective material modification in complex substances, by selectively affecting chemical bonds, inducing controllable surface temperature and heating rates that cannot be achieved by other methods. An example of such processing is the laser ablation of nitride ceramics. These are hard materials with excellent electrical insolating properties, with high thermal and chemical stability. The laser processing of these materials may result in the realization of decomposition reactions where the nitrogen can be ejected [14]. In the case of AlN ceramic, this will lead to the formation of an aluminium layer on the ceramic surface. In previous works we showed that this layer is conductive, as its conductivity can be modified by the processing conditions [15,16]. Furthermore, the formed layer usually has a nanostructure morphology which makes it a candidate in plasmonic applications.

Ammonium nitrate (NH_4_NO_3_) is a white crystalline salt than consists of ions of ammonium and nitrate. It is highly soluble in water and hygroscopic as a solid, although it does not form hydrates. It is predominantly used in agriculture as a high-nitrogen fertilizer (33.5 percent nitrogen), or as a component of explosive mixtures used in mining, quarrying, and civil construction. However, many countries are phasing out its use in consumer applications due to the concerns over its potential for misuse. The global production was estimated at 16.7 million tons in 2021. Nitrate and nitrite can be a risk for the production and supply of healthy drinking water. The standard of 50 mg/L NO_3_ in the Nitrates Directive is a derived value that protects the quality of drinking water [16].

Ammonium nitrate has been studied widely with Raman spectroscopy. Diaz and Hahn investigated pure ammonium nitrate and some mixtures, as the relative limit of detection of in water was 0.1% (1 mg/g), equivalent to an absolute limit of detection 1.0 μg [17]. Byram et al. [18] used Au nanostructures as SERS substrates, which were fabricated using ultrafast laser ablation in liquid as the detection limit achieved was 10^−5^ M. In [19], the authors produced silver nano-dendrite substrates and a lower concentration of 1 μM ammonium nitrate was detected, while the enhancement factor (EF) achieved was about ∼10^4^. Furthermore, Gajaraj et al. [20] used a commercially available Au nano-substrate (a gold-coated silicon material) and reported nitrate detection limits in water and wastewater samples of about 0.5 mg/L, at EF of the same value. In [21], a Raman sensor with 532 nm excitation was used for a metrology of inorganic substances as nitrate, sulphate and phosphate salts diluted in water. Specifically, Raman spectra of aqueous solutions of nitrate and nitrite were recorded. Nowadays, combining laser and ion implantation techniques [22,23], Ag/ZnO nanocomposites were applied for SERS detection of ammonium nitrate at different concentrations. Furthermore, the SERS method for measuring nitrate nitrogen in aquaculture water was developed using a substrate of β-cyclodextrin-modified gold nanoparticles (SH-β-CD@AuNPs) [24]. The femtosecond laser ablation technique was applied to fabricate silver-doped titanium dioxide nanoparticles, which were employed in surface-enhanced Raman scattering-based sensing of RDX and ammonium nitrate traces [25].

Resuming, analyses and detection of ammonium nitrate using Al structures has not been reported so far.

In this work, results on the application of laser-structured AlN ceramic surfaces in Surface-Enhanced Raman Spectroscopy of ammonium nitrate are presented. They present a new step in the field of detection of nitrate by using aluminium structures in SERS. Although, aluminium structures are considered as efficient plasmonic material in UV, here, we explore their properties in the visible spectral range, where most commercial Raman systems work. The study gives a new direction of use of AlN ceramics, as the data can be used in the development of complex integrated analytical systems including SERS active parts. It is also demonstrated that the use of the presented structures offers better efficiency, compared to areas fabricated in Al at the same processing conditions.

## 2. Materials and Methods

### 2.1. Materials and Laser Processing

The structuring of 0.6 mm-thick AlN ceramic surface plates (CeramTec, Plochingen, Germany) with a surface roughness of about ≈0.5 μm was accomplished with a Nd:YAG laser (Lotis TII, Minsk, Belarus) operating at 1064 nm, with a rep rate of 10 Hz, and pulse duration of 15 ns. The laser radiation was focused on the sample surface by a lens with a focal length of 300 mm. The ceramic surface was placed before the focal spot, as the beam spot size on the surface was 3 mm. The samples were placed in a vacuum chamber, which allows experiments in air at ambient pressure, and at pressures down to about ≈10^−^^4^ Torr. In this work, ablated spots fabricated in air at atmospheric pressure and at 1 × 10^−^^4^ Torr (referred as vacuum in the following text), at different laser fluences and number of applied pulses were studied.

### 2.2. Raman Scattering Measurements

An equal amount of water solution of ammonium nitrate was dropped by a pipette at different concentrations on the ablated zones. This was also performed on the native, non-processed ceramic surface and on glass. After drying of the liquid, Raman spectra were taken. Two types of Raman systems were used in order to estimate the efficiency of the fabricated structures: μ-Raman spectrometer (Renishow InVia, Wotton-under-Edge, UK) at excitation of 514 (3 mW) and at 785 nm (30 mW). Spectra from three positions within a spot were obtained, as for each spectrum, 10 scans were accumulated. Experiments with aluminium plate were also performed including laser processing and SERS experiments in order to estimate the benefits of using AlN target (Carrollton, TX, USA).

### 2.3. Materials Characterization

The samples’ surface morphology analysis was carried out by scanning electron microscopy (SEM) (FEI Quanta FEG 250 and Zeiss EVO 15, equipped with Energy Dispersive X-ray (EDX) spectrometer (Oxford Instruments, Abingdon, UK)); the phase and chemical characterization of the processed areas was based on EDX and X-ray photoelectron spectroscopy (XPS) (AXIS Supra electron spectrometer, Kratos Analytical Ltd., Stretford, UK) with the standard deconvolution software (ESCApe^TM^ 1.2.0.1325 of Kratos Analytical Ltd., Manchester, UK) analyses. The analysis of the obtained results is based on the calculations of the near-field enhancement at different conditions based on Finite Difference Time Domain simulation (Omnisim, Photon Design, Edmonton, AL, Canada) and the extinction spectra calculations based on Generalized Mie scattering theory [26,27]. The structures considered in these simulations were taken from the SEM images of the real samples.

## 3. Results

### 3.1. Laser-Induced Morphology Changes

**Figure 1:**
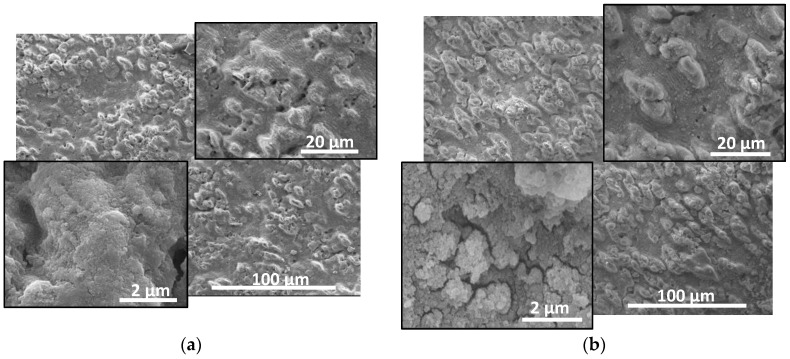

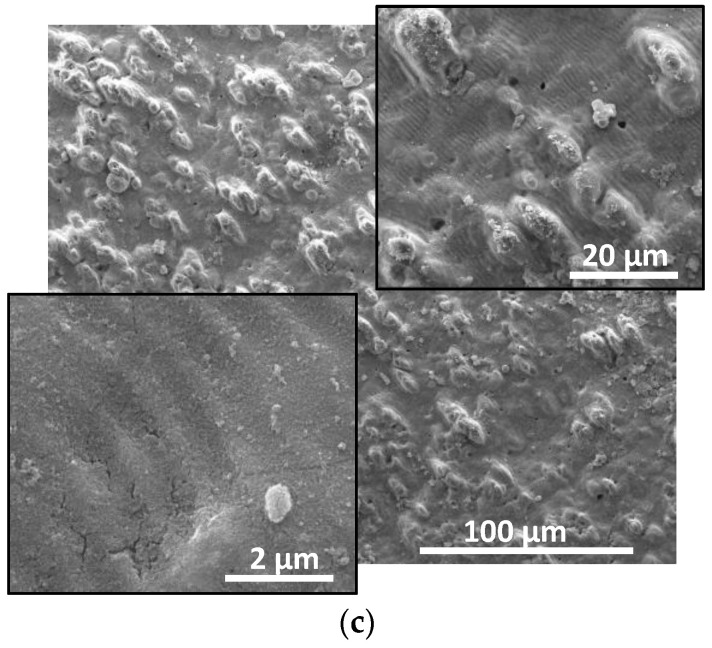
SEM images of the ablated areas in AlN ceramic in vacuum at: (**a**) laser fluence of 12 J/cm^2^, 100 laser pulses; (**b**) 18 J/cm^2^, 300 laser pulses; and (**c**) 20 J/cm^2^, 300 pulses. Images at different magnifications are given.

**Figure 2:**
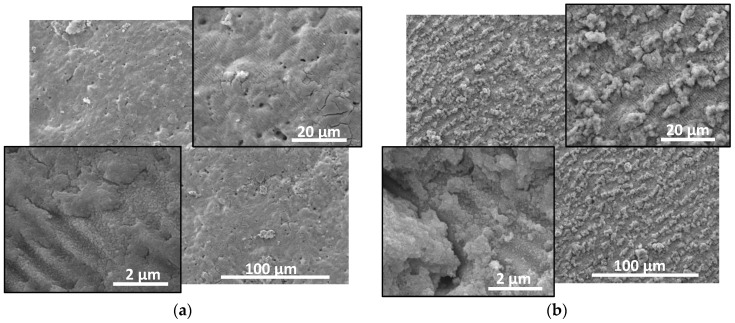

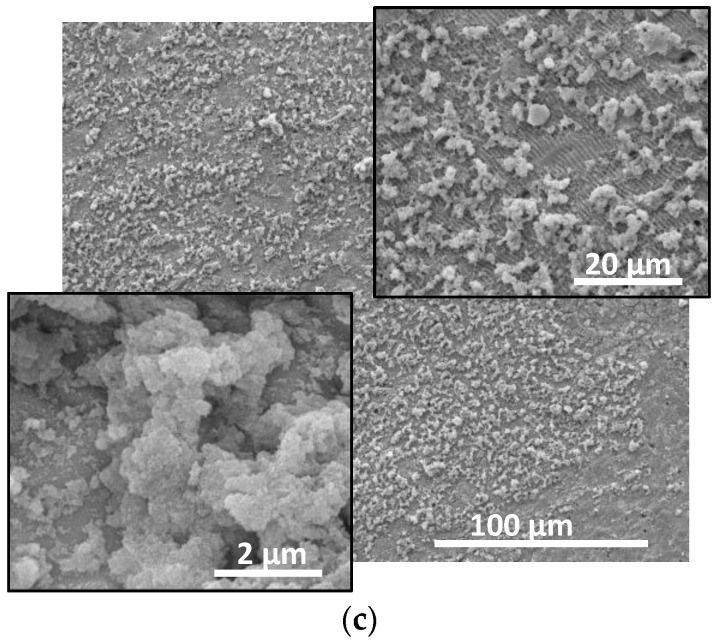
SEM images of the ablated areas in AlN ceramic in air at atmospheric pressure at: (**a**) laser fluence of 12 J/cm^2^, 100 laser pulses; (**b**) 18 J/cm^2^, 300 laser pulses; and (**c**) 20 J/cm^2^, 300 pulses. Images at different magnifications are given.

The native AlN ceramic used in this study has a grey matte colour. It is composed of crystallites with a typical size of few micrometres. The material is a good electrical isolator. The laser processing induces significant change in the properties of the irradiated zone. When ablation of the material is realized, this zone expresses a metal-like colour. The colour of the zones is different when the ablation is performed in vacuum or in air, which suggests there are different properties of the material. Figure 1 and Figure 2 show SEM images of ablated spots in AlN at different conditions in vacuum and in air, respectively.

The ablation process induces a complex morphology of the affected zone, as different micro- and nanostructures can be seen. In the case of processing in vacuum, all the surface is covered by micro-sized bumps that at the higher fluences used are elongated perpendicularly to the laser polarization. The effect is not clearly observed for processing at the lowest laser fluence used and with a pulse number of 100. The experiments performed indicate that with the increase in the applied pulse number, the formation of bumps with an elongated shape in the direction perpendicular to the laser polarization can also be formed. A comparison between images in Figure 1b,c shows that with the increase in the laser fluence, the number density of bumps decreases. The higher magnification images presented in Figure 1, show a formation of periodic structure, that could be referred to as ripples that are often observed in laser processing of different materials usually with ultrashort laser pulses.

Although such structures are typical when ultrashort laser pulses (pulse duration of pico- and femtoseconds) are used, here they also show the specific features already described [28]—they are with an orientation perpendicular to the laser polarization and the characteristic period is in the order of the used wavelength (here, the former is about 900 nm). The surface of the fabricated material has also a sub-micrometre structure as it can be seen from the images with the highest magnification. It is composed of nanoparticles with sizes in the range 10–100 nm, as in the case of processing at 18 J/cm^2^ and 300 laser pulses, they form a highly porous 3D structure (Figure 1b; in the other two cases shown in Figure 1a,c, the surface is smoother, which is clearly expressed for the case of using the highest fluence of 20 J/cm^2^ and application of 300 pulses (Figure 1c)).

The surface morphology differs when the laser processing is performed in air. The bumps with soft edges seen in the case of ablation in vacuum are not present here. In the case of irradiation at fluence of 12 J/cm^2^ and pulse number of 100 (Figure 2a), the surface is rather smooth. For the other processing conditions presented in Figure 2, the surface is decorated by micro-sized clusters of nanoparticles. In the case of processing at fluence of 18 J/cm^2^ and application of 300 laser pulses, these clusters are organized in lines oriented *parallel* to the laser polarization. This orientation is lost when the laser fluence is enhanced to 20 J/cm^2^ (Figure 2c). In all cases, ripple structures with the same characteristics as presented in the case of irradiation in vacuum are formed.

### 3.2. Material Composition

The material formed after the laser ablation in all cases presented is electrically conductive. By using two probe measurements, it was found that the resistance value is in the order of tens of Omhs. A higher value of about 10% is obtained for the structures fabricated in air. In order to clarify the chemical composition of the material in the irradiated zone, XPS analyses are performed.

**Figure 3:**
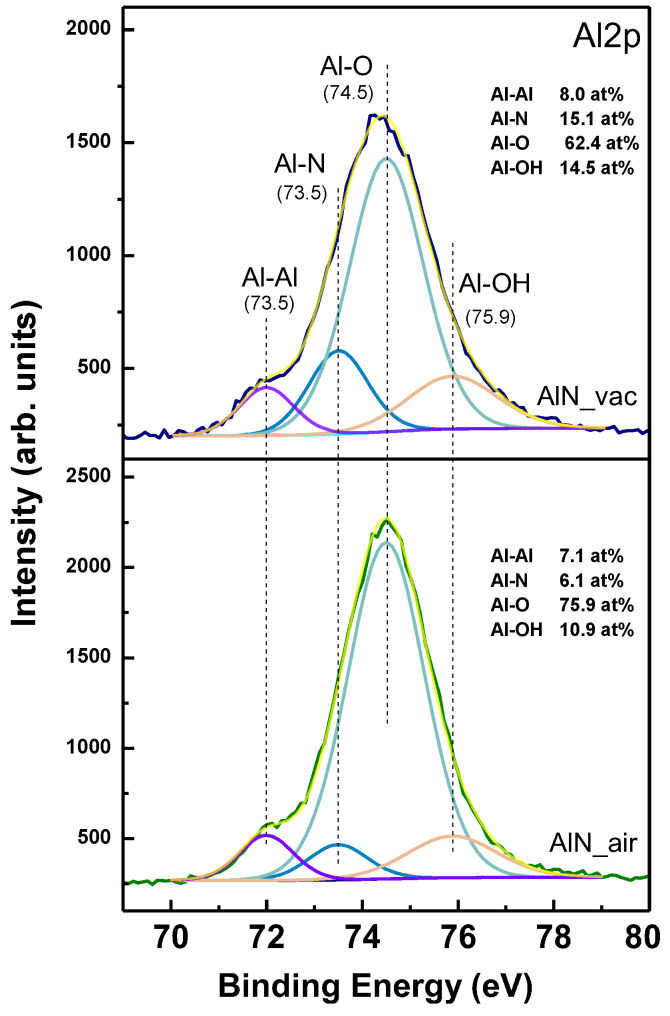
XPS spectra of Al2p for areas fabricated by laser ablation at 18 J/cm^2^ and by 300 laser pulses. (**top** image) Spectra when processing is in vacuum; (**bottom** image) spectra when processing is in air.

Figure 3 presents Al2p XPS spectra of the material at two ablation zones formed at the same laser parameters, but in vacuum (upper graph) and in air at atmospheric pressure (bottom). The obtained spectra are deconvoluted by the integration in the system software. In both cases, the presence of metallic aluminium is detected. The ablation results in the formation of oxide that dominates the surface composition in both vacuum and air conditions. As expected, the concentration of oxide in the sample processed in air is higher. It should be mentioned again that both structures are conductive, which means that the oxide layer has a thickness comparable with that of the native oxidation of Al (few nm). However, since the resistance value for the case of structuring in air is about 10% higher, one can conclude that the thickness in this case is also higher. This could explain the higher concentration of aluminium oxide for the sample processed in air seen in XPS analysis.

**Figure 4:**
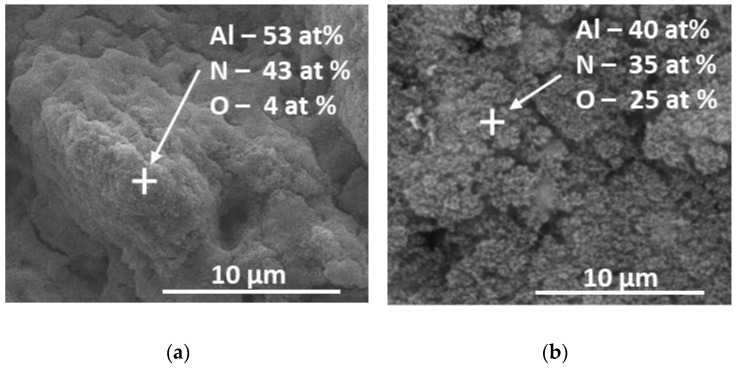
SEM images with marked areas where EDX analyses are performed. The laser fluence used is 18 J/cm^2^ and the applied pulse number is 300. (**a**) Ablation process is performed in vacuum; (**b**) ablation is performed in air at atmospheric pressure.

Further analysis of the surface composition is performed by EDX, which allows for estimation of the material composition in a limited area. Figure 4 shows the obtained compositions of the material in a specific area, which is indicated by a cross in the presented SEM images. In (a), data for material obtained by ablation in vacuum is given, and in (b) for the case of air. The laser fluence is 18 J/cm^2^ and the applied pulse number is 300 in both cases.

The estimation of the chemical composition given by EDX analysis clarifies that the clusters observed on the surface of the material processed in air (Figure 2b,c) contains nanoparticles with a high composition of aluminium oxide.

### 3.3. Application in SERS

**Figure 5:**
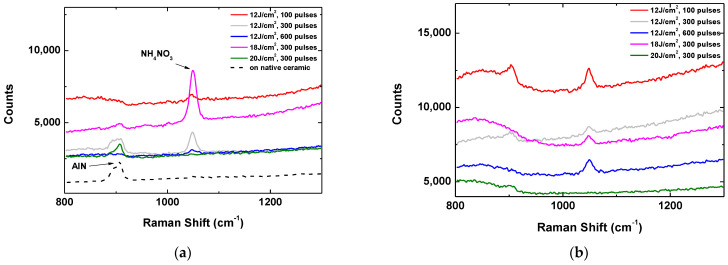
Raman spectra of NH_4_NO_3_ deposited on structures fabricated on the surface of AlN ceramic at different laser parameters. (**a**) Laser structuring is performed in vacuum; (**b**) laser structuring is performed in air at atmospheric pressure.

The above presented results indicate that laser ablation of AlN ceramic leads to the formation of aluminium structures on the surface. These include nanostructures which may result in an efficient excitation of plasmons and enhancement of the electromagnetic field in the close vicinity of the material’s surface. These properties can be used in applications as SERS. Figure 5 represents Raman spectra of ammonium nitrate (NH_4_NO_3_) deposited on structures obtained at different processing conditions in vacuum (a) and in air (b), respectively. The concentration of the analysed material is 500 mg/L. The Raman spectra are taken at an excitation wavelength of 514 nm.

The spectrum taken from a spot on the native surface of the ceramic with deposited ammonium nitrate is also present. It is seen that the Raman peak corresponding to the nitrate is not detected in this case. The strongest signal for the case of processing in vacuum is obtained for a structure fabricated at a fluence of 18 J/cm^2^ and with an applied pulse number of 300. With the same number of laser pulses, processing at a higher fluence of 20 J/cm^2^ results in a structure that does not enhance the Raman signal and the peak of NH_4_NO_3_ is not detected. For the structure obtained at a fluence of 12 J/cm^2^ and 300 laser pulses, the enhancement of the Raman signal is observed as it is about twice as low compared to the case of processing at 18 J/cm^2^. As is seen from Figure 5, the optimal structure for maximal enhancement of the Raman signal obtained in air is different than in vacuum. In the used experimental conditions, the highest signal is observed for the structure fabricated at a laser fluence of 12 J/cm^2^ and the application of 100 laser pulses. The intensity of the Raman peak of ammonium nitrate obtained with this structure is about two times lower compared to the maximal one, measured for the optimal structure fabricated in vacuum.

**Figure 6:**
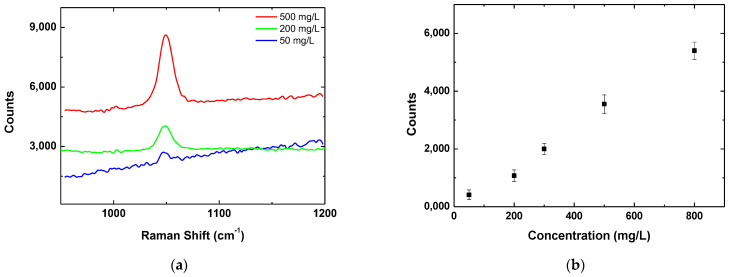
(**a**) Raman spectra of NH_4_NO_3_ at different concentrations deposited on structure fabricated at a fluence of 18 J/cm^2^ and with an applied pulse number of 300 in vacuum; (**b**) dependence of the Raman peak intensity (at 1049 cm^−1^) on the concentration of the ammonium nitrate. The bars show the variation of the value from three measurements at different positions.

In order to estimate the detection limit (the lowest detectable concentration of NH_4_NO_3_), the optimal structure fabricated in vacuum is used. On three identical spots, ammonium nitrate is deposited at different concentrations. Figure 6a shows the Raman spectra for these cases. The results indicate that the presented structures can be used for the detection of ammonium nitrate at concentrations lower than 50 mg/L. In Figure 6b a dependence of the Raman peak intensity on the ammonium nitrate concentration is presented. Information about the variation of the value from three measurements from different positions is given by the error bars. It shows that this variation does not exceed 20% which can be an estimation of the reproducibility of the results.

**Figure 7:**
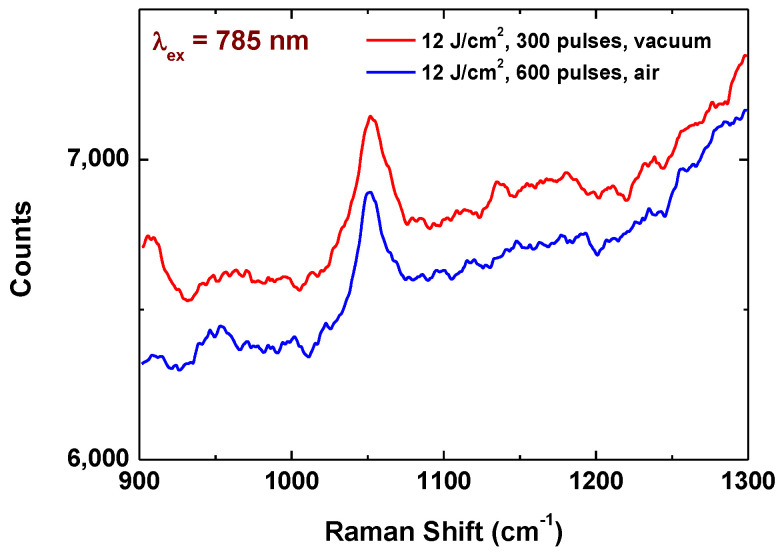
Raman spectra of NH_4_NO_3_ deposited on structure fabricated at different conditions. The excitation wavelength is 785 nm.

Figure 7 represents the Raman spectra on NH_4_NO_3_ at a concentration of 500 mg/L, taken at an excitation wavelength of 785 nm. Since at these conditions Raman peaks from the nitrate are not detected when it is deposited on native ceramic surface, one can assume that at this wavelength the structure also induces enhancements of the signal. The intensity of the nitrate peaks are about six times lower compared to that at optimal conditions when excitation is at 514 nm. It should be mentioned that the most efficient structures when excitation is at 785 nm do not correspond to these when the excitation wavelength is 514 nm.

In order to quantify the efficiency of the fabricated structures, the enhancement factor is estimated. The relation [29]:

EF = (I_SERS_/I_R_). (N_R_/N_SERS_), (1)

is used, where I_SERS_ and I_R_, are the intensities of strongest detected peak when the aluminium structure is considered and on the native ceramic surface, respectively. N_SERS_ and N_R_ are the number of molecules that contribute to the corresponding signal. In this work, the same amount of ammonium nitrate solution is deposited on the aluminium structure and on the native ceramic surface, so one can consider that N_R_/N_SERS_ ≈ 1, and the enhancement factor is given by the relation between the intensities of the measured Raman peak. The estimation performed for the ammonium nitrate line at 1049 cm^−1^ gives EF of 6 × 10^4^ at 514 nm, and 8 × 10^3^ at 785 nm, as the best values.

**Figure 8:**
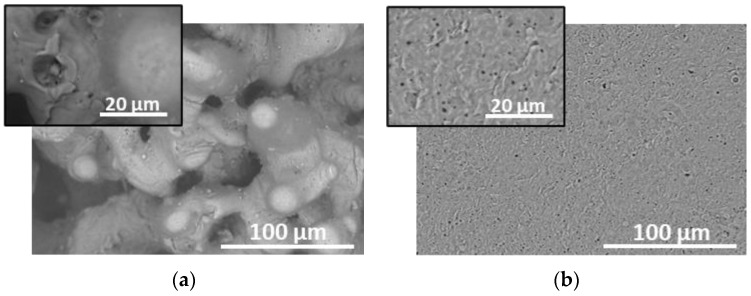
SEM images of areas ablated in Al at 18 J/cm^2^ and application of 300 laser pulses. (**a**) Ablation in vacuum; (**b**) ablation in air.

For comparison, the efficiency of structures fabricated by laser ablation of Al plate are also investigated. Figure 8 shows the SEM images of the ablated area at 18 J/cm^2^ and application of 300 laser pulses in vacuum (a) and in air (b). In the case of processing of Al at the presented conditions, the formed morphology is different than the case of AlN ceramic (Figure 1 and Figure 2). Here, ripple structure and nanoparticle and nanoparticles clusters are not observed in both cases of ablation in vacuum and in air. It should be mentioned that for these structures the Raman signal of ammonium nitrate is not detected at concentrations lower than 100 mg/L at an excitation wavelength of 514 nm.

## 4. Discussion

### 4.1. Ablation Mechanism

The laser ablation process of AlN ceramic by nanosecond pulses is realized by material decomposition. A different reaction may take place on the surface depending on the applied laser fluence. The decomposition reaction that requires minimal energy includes the ejection of nitrogen from the surface and the formation of a layer composed of aluminium, respectively, according to the reaction [30]:

AlN → Al_(l)_ + 1/2N_2(g)_, (2)

The nitrogen pressure increases rapidly with the temperature, as above the decomposition point (about 2600 K) [31], it could induce violent material removal. The performed experiments use a multi-pulse regime, where the next pulse faces the aluminium layer on the surface. Its ablation includes evaporation due to heating above the evaporation temperature. Furthermore, the violent ejection of nitrogen at high pressure would result in a vast expansion of the liquid metal that leads to formation of aluminium nanoparticles. Such particles and their clusters are usually seen in the SEM images presented in Figure 1. The formation of ripple structures presented in the ablated zones can be attributed to interference of the incident and the scattered field [15,28], as this model correctly describes the period and orientation of the periodic structures observed. The formation of the elongated bumps could be described as complex phenomena that includes spatial selective ablation related to morphology influenced absorption, near field intensity localization and polarization dependent reflection [15]. The morphology of the ablated areas fabricated in vacuum and in air is different as is seen from Figure 1 and Figure 2. The reason can be found in the enhanced effect of redeposition of ablated material in the case of ablation in air. The presence of redeposited species changes the interaction between the laser radiation and material, influencing absorption and scattering. As it is mentioned above, these strongly affect the mechanisms that play roles in the formation of surface morphology. In addition, the oxidation process will also affect not only the surface composition but also the morphology. The formation of an oxide layer may influence the optical properties of the surface, i.e., the absorption of the laser radiation. Furthermore, the process of oxidation is an exothermic reaction, where the released heat may lead to local modification of the surface morphology and composition. As is shown in the analyses presented in both cases of ablation in vacuum and in air, the surface of the material is oxidized. In the case of vacuum ablation, this oxide is formed after the ablation process, when the material is cooled to the room temperature. In the case of ablation in air, the process is realized during the cooling of the surface. Since the efficiency of oxidation is proportional to the temperature, the presence of oxide phase is more pronounced for the ablation in air. Due to this the clusters of nanoparticles seen in the SEM images in Figure 2 are composed mainly of aluminium oxide.

### 4.2. Optical Properties

**Figure 9:**
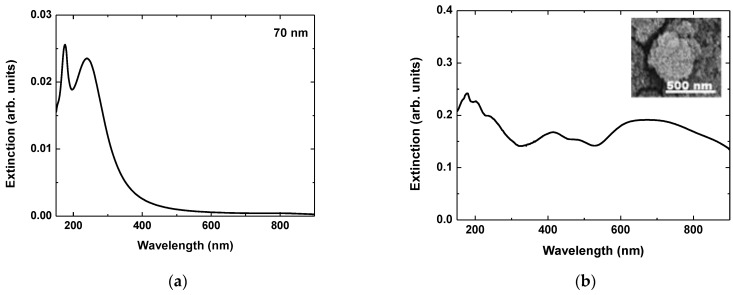
Calculated extinction spectra of: (**a**) single isolated spherical Al nanoparticle with diameter of 70 nm; (**b**) cluster composed of 650 particles with a diameter of 70 nm. The size of the particles and the cluster geometry are estimated from the SEM image of the structure fabricated at a fluence of 18 J/cm^2^ and the application of 300 pulses inserted in (**b**).

The efficiency of the application of a structure in SERS depends mainly on its ability to enhance the electric field intensity in its near field following the relation I_SERS_~IEI^4^ [2]. Different mechanisms may contribute to such enhancement. In the case of metal nanostructures, the efficient excitation of plasmons results in a strong enhancement of the electric field intensity [32]. The effect can be enhanced dramatically when the system is composed of nanostructures separated by a nanosized gap. In this case, plasmon coupling is realized as the electric field intensity may be enhanced by orders of magnitude compared to the incident one. A different mechanism of field intensity enhancement can be related to the “lightening rod effect” where charge accumulation is observed in sharp edges of conductors. In the case of plasmon-based field enhancement, the efficiency strongly depends on the incident wavelength since plasmon excitation is a resonance effect. The resonance wavelength depends on the structure’s characteristics—shape, size, morphology. In the case of Al, isolated nanoparticles have plasmon resonance in the UV spectral range, however, when ensembles of nanoparticles or nanoparticles with sizes of few hundreds of nanometres are considered, the resonance broadens and it is red shifted. In order to estimate this effect, Generalized Mie scattering theory is applied. Figure 9 represents extinction spectra of a single isolated nanoparticle with diameter of 70 nm, and a cluster composed of 650 Al nanoparticles with a diameter of 70 nm. The simulation results indicate that for the cluster, the extinction spectrum is very broad, covering the visible spectral range.

**Figure 10:**
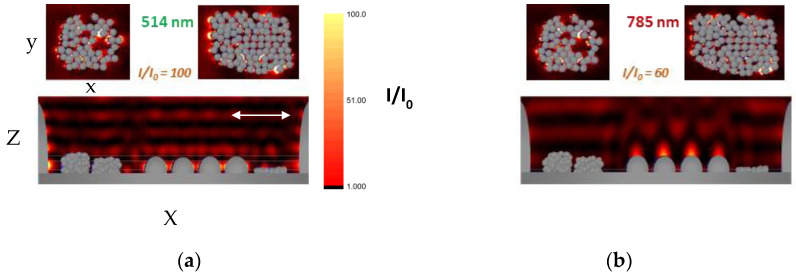
Distribution of the electromagnetic field in the vicinity of Al structure obtained by FDTD simulation. The structure consists of bumps placed in the edges of the simulation system; ripple structure with crests width of 600 nm and distance between them 150 nm; and 2D nanoparticle array and two nanoparticle 3D clusters. The nanoparticle array is composed of randomly distributed 90 nanoparticles. The nanoparticle size is 70 nm. The nanoparticle clusters are composed of 130 and 200 nanoparticles, respectively. The intensity enchantment I/I_0_ is given for the different cases. (**a**) the excitation is at 514 nm; (**b**) at 784 nm. The white arrow in (**a**) indicates the polarization of the incident wave. It propagates in the Z direction. The distribution of the intensity is given in a plane through the equators of particles in the 2D array (up right in (**a**,**b**)) and in a plane through the equators of the particles from the top layer in the larger cluster (up left in (**a**,**b**)).

In order to estimate the efficiency of enhancement of the near electromagnetic field in the vicinity of the fabricated structures, FDTD simulations are performed. As is seen from the SEM images, the surface morphology of the ablated areas is characterized by three main components—micro-sized bumps, ripple structures and nanoparticles and nanoparticle clusters. In FDTD simulation, a real part of the surface of the ceramic processed at fluence of 18 J/cm^2^ and application of 300 laser pulses is considered. The simulation considers the above-mentioned Al structures irradiated by a linearly polarized plane wave. Calculations are performed for the two wavelengths 514, and 785 nm, that corresponds to the excitation sources of the real Raman systems used. The simulation system is 3D with a size 3 × 3 × 9 µm^3^ divided into cubic cells with a size of 5 nm. For simulation, the dielectric properties of Al included in the software are used. They are taken from [33]. Figure 10 shows the electromagnetic field distribution for the three wavelengths. The simulation results show that the intensity enhancement as a ratio between the developed intensity, I, and the incited one, I_0_, is different at the different excitation wavelengths. The dependence corresponds to the dependence of the enhancement factor for the different wavelengths. Thus, one can explain the different efficiency by different enhancements of the near field intensity. Furthermore, in the case of excitations at 514 nm, the zones with the highest intensity enhancement (hot spots) are located between the nanoparticles. About an order of magnitude lower, enhancement is observed in the gap between crests of the ripple structure. The number density of hot spots decreases at an excitation of 785 nm. However, here, large areas with intensity enhancement are formed on the top of the ripple’s crests. The intensity value is higher than in the case of excitation at 514 nm as it is similar to that in the hot spots in the nanoparticle structures. These results indicate that SERS activity of the presented Al structure can be realized at a wide range of Raman excitation wavelengths, but the hot spots’ spatial distribution and the mechanism of enhancement of the near field could be different. As Figure 9b shows, plasmon excitations could be efficiently induced in a broad spectral range. These are mainly responsible for the field enhancement in the nanoparticle arrays. In the case of excitation at 785 nm, the “lightening rod effect” can be considered to play a role in the structure performance. It should also be mentioned that for a simulation performed when bumps were not present, lower values were given for the field intensity with a factor of 1.5.

Although aluminium structures based on decomposed AlN ceramics are not used in SERS so far, some results of this work can be compared to those already published in the field. Al structures were used in SERS at different wavelengths ranging from UV to the visible spectral range [11]. The reported enhancement factor is in the range 10^3^–10^6^. In [34], the authors used Al nanodot obtained by the deposition of Al on a pre-structured surface for SERS of triethoxyphenylsilane and naphthalene. The used pump wavelength was 532 nm. The authors report the SERS effect, as the enhancement factor estimated was ×10^4^. Al nanostructures fabricated by laser ablation are also used for SERS at 532 and 785 nm excitation, for the detection of crystal violet and Rhodamine6G [9]. The lowest detectable concentration reached is in the femtomolar range. We should mention however that in most of the cases, active substrates are fabricated by lithography or chemical methods that are less efficient in terms of cost and production speed compared to those presented here. It also important that the efficiency of the application of Al structures in SERS is strongly analyte-selective, since those formed on the surface oxide layer may benefit from binding to different functional groups. In the case presented here regarding of ammonium nitrate, SERS efficiency was lower when the AlN was processed in air. Since the hot spots are mainly localized between nanoparticles, oxidation will induce an increase in the gap between the nanoparticles which leads to less efficient plasmon coupling and the decrease in the nearfield intensity [32]. The lower efficiency of the structure fabricated by ablation of Al plate could be explained by the absence of nanoparticles and nanoparticle clusters, which are the source of the hot spots with the highest field enhancement, as shown in Figure 10.

## 5. Conclusions

This work demonstrates the fabrication of an Al structure by laser ablation of AlN that can be used in SERS. It is demonstrated that at certain conditions the detection of ammonium nitrate at concentration of 50 mg/L (which is the maximal allowed in drinking water) can be achieved. The presented structures can be used in SERS at different excitation wavelengths. The theoretical analysis demonstrates the formation of zones with a strong enhancement of field intensity, as its spatial distribution has different features for the different wavelengths. This finding can be used for the optimization of the SERS performance. The obtained data can be applied for the development of complex analytical systems based on AlN ceramics, but also can be a basis for further understanding of the relation between the electromagnetic field properties in the near field zone and Raman signal enhancement.

## Data Availability

The raw data supporting the conclusions of this article will be made available by the authors on request.
